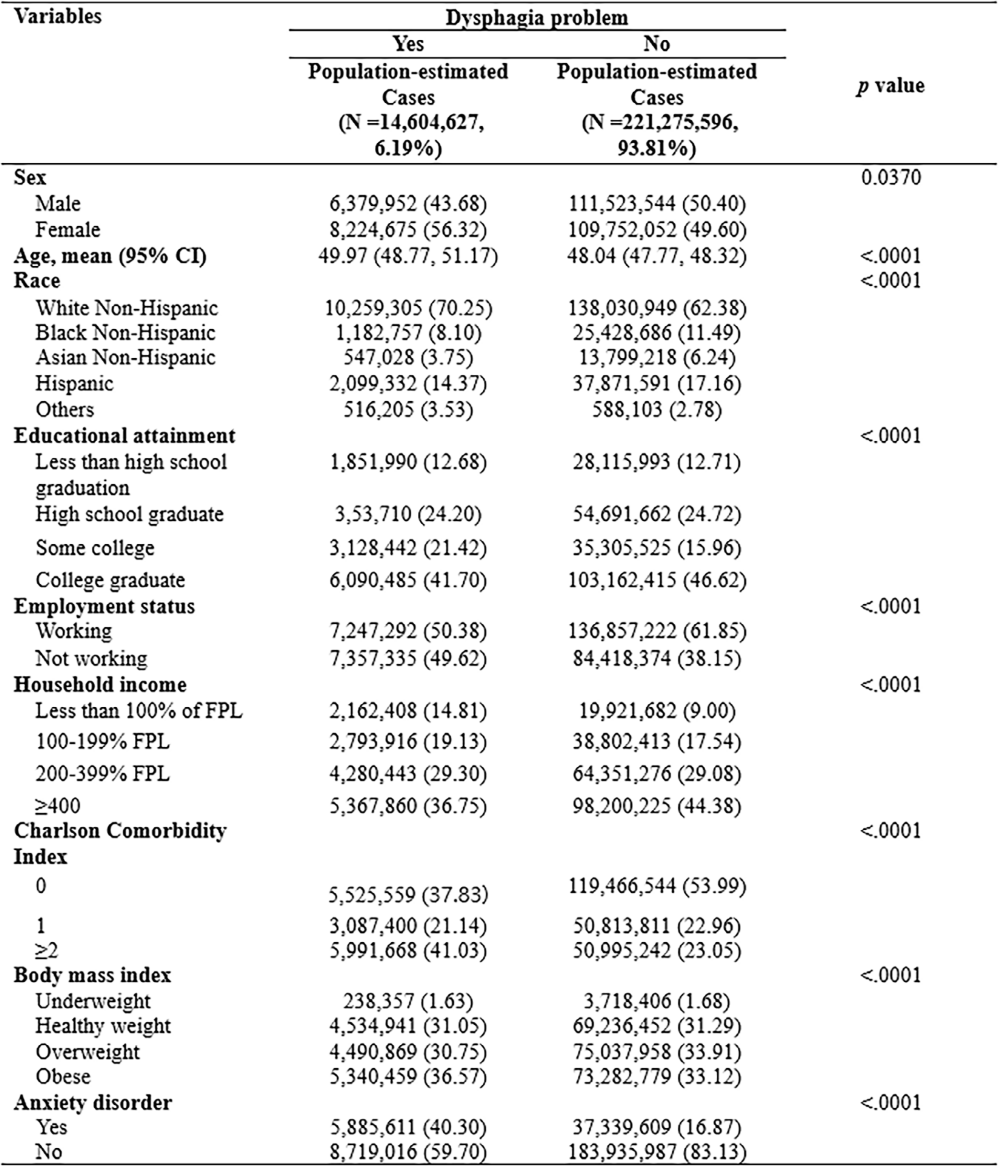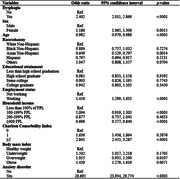# Uncovering the association between dysphagia and depression: Insights from the 2022 National Health Interview Survey

**DOI:** 10.1002/alz.088631

**Published:** 2025-01-03

**Authors:** Sanghee Yoo, Ickpyo Hong

**Affiliations:** ^1^ Yonsei university, Wonju, Gangwon‐do Korea, Republic of (South); ^2^ Yonsei University, Wonju, Gangwon‐do Korea, Republic of (South)

## Abstract

**Background:**

Dysphagia can increase fear of swallowing, reduce self‐esteem, and hinder social relationships. Such factors can also increase anxiety and degrade the quality of life. This study aimed to elucidate the association between dysphagia and depressive symptoms.

**Method:**

This study conducted an assessment of 26,810 U.S. adults using the 2022 National Health Interview Survey (NHIS) database. Multiple regression analysis and five different propensity score matching approaches were used to account for sex, race/ethnicity, educational attainment, household income, number of comorbid chronic diseases, and presence of anxiety disorder.

**Result:**

Utilizing the 2022 NHIS database, 26,810 American adults were assessed. Of the weighted population demographics, 51.88% reported having dysphagia, with 43.64% being females and an average age of 46.17 (SD 0.22). The study findings revealed that individuals reporting dysphagia had an elevated likelihood of presenting depressive symptoms (OR = 1.875, p < .0001). Several factors influencing depressive symptoms were identified, including sex, race, educational attainment, household income, the number of accompanying chronic diseases, and the presence of an anxiety disorder. Females exhibited a higher likelihood of depression (OR = 1.441; 95%CI 1.354, 1.533) compared to males. Individuals with educational levels of ‘some college’ or higher demonstrated elevated odds ratios for depressive symptoms relative to those who did not complete high school (OR = 1.141; 95%CI 1.018, 1.279). The presence of two‐plus chronic diseases significantly upped depression risks (OR = 1.902; 95%CI 1.725, 2.097), with anxiety disorders further amplifying this (OR = 5.147; 95%CI 4.676, 5.664).

**Conclusion:**

This study demonstrated the intricate interplay between dysphagia and various demographic and health factors, emphasizing the critical need for individualized management strategies for depression in patients with dysphagia.